# Exploring the Implicit Link Between Red and Aggressiveness as Well as Blue and Agreeableness

**DOI:** 10.3389/fpsyg.2020.570534

**Published:** 2021-01-15

**Authors:** Lu Geng, Xiaobin Hong, Yulan Zhou

**Affiliations:** ^1^Department of Psychology, Wuhan Sports University, Wuhan, China; ^2^Hubei Key Laboratory of Exercise Training and Monitoring, College of Health Science, Wuhan Sports University, Wuhan, China

**Keywords:** color, stroop task, implicit association test, red, aggressiveness-agreeableness, blue

## Abstract

Previous studies have found a link between red and aggressive behavior. For example, athletes who wear red uniforms in sports are considered to have a competitive advantage. So far, most previous studies have adopted self-report methods, which have low face validity and were easily influenced by the social expectations. Therefore, the study used two implicit methods to further explore the association between red and aggressiveness. A modified Stroop task was used in Experiment 1 to probe college students’ differences between “congruent” tasks (i.e., red–aggressiveness and blue–agreeableness) and “incongruent” tasks (i.e., red–agreeableness and blue–aggressiveness). Result showed that participants responded more quickly to the congruent tasks than the incongruent tasks. Then, in order to adapt to the competitive context, Experiment 2 used an implicit association test with photos of athletes as the stimulus to college students and athletes to evaluate “congruent” tasks (i.e., red uniform photo-aggressiveness and blue uniform photo-agreeableness) as well as “incongruent” tasks (i.e., red uniform photo-agreeableness and blue uniform photo-aggressiveness), respectively. According to the results, both college students and athletes respond faster to congruent tasks than to incongruent tasks. Besides, athletes’ reactions to the red–aggressiveness association are faster than college students, which may relate to the athletes’ professional experience. The athletes may be more aggressive and impulsive. Overall, the study has attempted to examine the association between red and aggressiveness through implicit methods, but in the future, researches are need to find a deep association from brain mechanism aspect.

## Introduction

Color perception is a common sensation in daily lives, not only as an esthetic awareness but also in terms of its wider impact on human psychology and behaviors. Theories about color and psychological functioning have been proposed since the early 19th century (Goethe, 1975, p.115), but only in the last decade, an increasing body of empirical researches began to document the influence of color on human consciousness and conduct. Aside from a few studies exploring the effect of black (Frank and Gilovich, 1988; Şefik Tiryaki, 2005; Caldwell and Burger, 2011), the majority of prior researches have examined the effect of red on psychological functioning, as cognitive performance (Mehta and Zhu, 2009), sexual attractiveness (Elliot and Niesta, 2008), financial prediction (Jiang et al., 2014), competitive sports (Hill and Barton, 2005), and food consumption (Bruno et al., 2013). Researchers have been interested in discerning advantage for competitors wearing red. In their analysis of the Olympic Games in 2004, Hill and Barton (2005) reported an advantage for athletes wearing red (vs. blue) in combat sports such as boxing, Taekwondo, and wrestling. They stated that the advantage conferred by red uniforms may be because of an evolutionary, engrained, and social learning association of red with dominance and aggression.

Hill and Barton (2005) provoked additional academic interest in testing the advantages of wearing red in competition, but inconsistent results have been obtained (Attrill et al., 2008; Sorokowski and Szmajke, 2011; Piatti et al., 2012; Curby, 2016), which indicated that various factors may affect athletes’ performance in competition, and red is not the only one. From an implicit perspective, most of the above results are archival research. Thus, the association between red and aggressiveness is possible. Other studies have focused on the benefits originated from the association between red and aggression. Little and Hill (2007) found that, compared to blue shapes, red shapes were regarded to be more aggressive and dominant and more likely to win in physical competitions.

Researchers also examined the influence of red on social perceptions of dominance and aggressiveness in non-competitive contexts. In one study, men in red were rated as more aggressive and more dominant than those in blue or gray (Wiedemann et al., 2015). Briki and Hue (2016) studied how red, blue, and green were judged in relation to conceptualizations of dominance, arousal, and pleasure and found that red is strongly associated with dominance. In competitive contexts, Feltman and Elliot (2011) confirmed that wearing red can enhance perceptions of the dominance and threat, both of the opponents’ and of one’s own. The factors that affect the performance include not only the players and opponents but also the referee (Plessner and Haar, 2006). Some studies explored the advantages of red uniform from the perspective of referee and found that red is more dominant and aggressive (Hagemann et al., 2008; Krenn, 2014, 2015).

Color-in-Context Theory holds that there are two sources in color meanings: learning and biology (Elliot and Maier, 2012). For example, in competitive context, red-headed birds were found to be more likely to win than black-headed or yellow-headed birds (Pryke and Griffith, 2006). In addition, red in a male mandrill or baboon’s face and genitalia is a symbol of status: the brighter the red is, the stronger the male’s attack power will be (Setchell and Wickings, 2005; Bergman et al., 2009). Human beings, as their closest primate relatives, hold the same color-meaning pairings as those animals have. Shi et al. (2015) indicated that viewing red can impair participants’ performance during a challenging cognitive task. This suggests that red is always associated with warning in the study context due to repetition of wrong pairings (e.g., teachers often mark errors with red pen). Social learning of red can also be extended to the national level. More recently, a study found that red was the most frequently used color in national flags across the world. This may because red was often attached with an aggressive connotation, and red can better reflect the competitiveness of the country. Compared to blue, red is rarely used in collaborative organizations (Zhang et al., 2018).

Associated with clear sky and clean water, blue is often used as the opposite color of red in competitive context. Blue represents peace and quiet in many cultures. It is noteworthy that blue also has different meanings in different cultures. Studies suggested that participants from the United States, Germany, and Turkey associate blue with positive meanings, thereby triggering positive emotions (Garth and Collado, 1921; Gesche, 1927; Choungourian, 1968). However, participants from Japan, the Philippines, Rome, Kuwait, and American India associate blue with negative connotations, which trigger negative emotions (Valdez and Mehrabian, 1994; Camgöz et al., 2002; Wogalter et al., 2002; Hurlbert and Ling, 2007; Frühholz et al., 2009; Gerend and Tricia, 2009). Associated with clear sky and clean water, blue is often used as the opposite color of red in the competitive context. Blue represents peace and quiet in many cultures. It is noteworthy that blue also has different meanings in different cultures. Studies suggested that participants from the United States, Germany, and Turkey associate blue with positive meanings, thereby triggering positive emotions (Garth and Collado, 1921; Gesche, 1927; Choungourian, 1968). However, participants from Japan, the Philippines, Rome, Kuwait, and American India associate blue with negative connotations, which trigger negative emotions (Valdez and Mehrabian, 1994; Camgöz et al., 2002; Wogalter et al., 2002; Hurlbert and Ling, 2007; Frühholz et al., 2009; Gerend and Tricia, 2009).

Above researches, it is proposed that there may be an implicit association between color and meanings. However, most studies adopted self-report methods, which have low ostensible validity and can easily be influenced by social expectation effect. Thus, the study used the implicit methods to explore the issue. Moreover, Jiang et al. (2014) found a “red up and green down” effect among Chinese mainland but a “green up and red down” among Hong Kong, which suggests that social culture might affect color and its associations. Does an association between red and aggressiveness exists among Chinese people? Is it consistent with the research carried out in the West? Experiment 1 assumed that, according to response competition logic (Klinger et al., 2000), if red is associated with aggressiveness, then the simultaneous presentation of red and aggressiveness-related words will be classified more quickly. Same trend applied for agreeableness-related words shown in blue.

Only a few studies conducted experiments from an implicit perspective (Moller et al., 2009; Soriano and Valenzuela, 2009; Fetterman and Meier, 2012; Pravossoudovitch et al., 2014). Mentzel et al. (2017) used a modified Stroop task to testify the red-dominance association, with lexical stimuli only. However, it is not specific in the competition context. Thus, Experiment 2 used the photo of athletes in red as the stimulus to examine the implicit association between red and aggressiveness. Based on the Five-Factor Model (FFM) of personality (Pervin and John, 1997); Trninić et al. (2008) found that the athletes have a significant correlation between aggressiveness and extraversion, and agreeableness and emotional stability. There are significant negative relations between emotional stability and aggressiveness. From the aspect of their development, adolescence is a turbulent life period; the athletes in the present study are young men in the adulthood late stage, and they are vulnerable to experience strong emotions. Experiment 2 postulated that the athletes will attain a shorter response time than college students in the association between red and aggressiveness.

In summary, the present study applied two implicit methods—a modified Stroop task and an implicit association test (IAT)—to explore red advantage and blue meaning among Chinese people.

## Experiment 1

In this experiment, aggressiveness-related words and agreeableness-related words are presented in red or blue; the aim of the experiment was to examine whether aggressiveness-related words presented in red would be categorized more quickly.

### Methods

#### Participants

Among 80 college students (43 females; mean age = 18.81 ± 0.86), all right-handed and not red–green colorblind, none took part in a similar psychological experiment before. To enhance the response rate, pre-paid envelopes were provided along with a small gift.

#### Stimuli and Pilot Test

In the modified Stroop task, ten Chinese words were used as lexical stimuli, five denoting aggressiveness (*bullying*, *offense*, *murder*, *aggression*, and *war*) and five denoting agreeableness (*close*, *caring*, *gentle*, *friendly*, and *harmonious*). All ten Chinese words consisted of two characters with the same word length. The words were rated in a pilot test by 97 college students according to the degree of aggressiveness, agreeableness, and familiarity, on a scale of 1 (*not at all*) to 5 (*extremely*). The aggressiveness-related words were rated as more aggressive (*M* = 4.58, SD = 0.71) than the agreeableness-related words (*M* = 1.24, SD = 0.49), *t* = −82.89, *p* < 0.001; the agreeableness-related words were rated as more agreeable (*M* = 4.48, SD = 0.77) than the aggressiveness-related words (*M* = 1.18, SD = 0.46), *t* = 79.185, *p* < 0.001. Moreover, all the words were rated as equal in terms of familiarity, *t* = 0.636, *p* > 0.05. The RGB criteria of red (255, 0, and 0) and blue (0, 0, and 255) were applied to the words. The HSL (hue, saturation, and lightness) criteria are as follows: red (0, 240, and 120) and blue (160, 240, and 120). The words constituted a 2 (Valence: aggressiveness vs. agreeableness) × 2 (Color: red vs. blue) lexical stimulus.

#### Design and Procedure

Special software E-prime 2.0 was used with a 12.1-inch screen Lenovo Think pad X200 for the stimulus presentation and data logging. After the experiment, the subjects were asked whether they could guess the purpose of the experiment, while all the subjects said no.

The experiment had a 2 (Valence: aggressiveness vs. agreeableness) × 2 (Color: red vs. blue) repeated-measures design. Each word was separately presented in red and blue on a black computer screen in random order. Participants were asked to press a key labeled to judge whether the word was aggressiveness-related or agreeableness-related. There were 20 trials in each practice block and 60 trials in the experiment block.

Before each word was displayed, a fixation cross appeared for 500 ms in the center of the computer screen. The word rendering time was 3,000 ms, and participants who did not make a response within the 3,000 ms went directly to the next trial. Participants whose response times were longer than 3,000 ms or shorter than 300 ms were eliminated from the analyses.

### Results and Discussion

**FIGURE 1:**
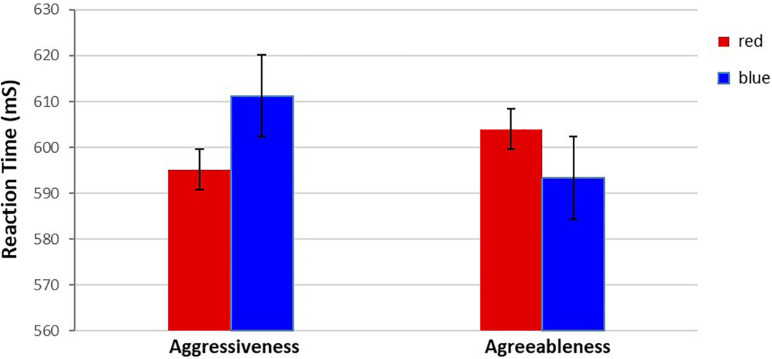
I Mean response times for aggressiveness and agreeableness words presented in red and blue.

A 2 (Color: red vs. blue) × 2 (Valence: aggressiveness vs. agreeableness) repeated-measures analysis of variance (ANOVA) regarding reactions times revealed a significant main effect of Color, but not Valence, *F*(1,75) = 27.63, *p* = 0.007 (*p* < 0.01), η^2^ = 0.03, with participants found to be faster in categorizing red words (*M* = 599.59 ms, SD = 124.13) than blue words (*M* = 602.26 ms, SD = 148.25). More importantly, a significant Valence × Color interaction, *F*(1,75) = 42.27, *p* = 0.041 (*p* < 0.05), η^2^ = 0.17, indicated that participants were faster in categorizing aggressiveness**-**related words presented in red (*M* = 595.16 ms, SD = 191.60) than in blue (*M* = 611.19 ms, SD = 202.19), *t*(76) = −1.46, *p* = 0.038 (*p* < 0.05), and were faster in categorizing agreeableness**-**related words presented in blue (*M* = 593.33 ms, SD = 116.82) than in red (*M* = 604.01 ms, SD = 191.54), *t*(76) = 3.92, *p* = 0.043 (*p* < 0.05) (see Figure 1).

Thus, the results indicated that red is positively associated with aggressiveness, because the aggressiveness-related words presented in red were categorized more quickly than in blue. In addition, blue was found to be positively associated with agreeableness, as agreeableness-related words presented in blue were categorized more quickly than in red.

## Experiment 2

In the experiment, the IAT (Greenwald et al., 1998) was used to test whether the college students and the athletes have differences in response time between congruent tasks and incongruent tasks.

The IAT included two tasks: congruent task and incongruent task. When the relationship between a concept and an attribute was consistent with participants’ implicit attitudes, it was considered congruent task. Participants typically follow their original attitude when making such judgments, which results in shorter response times. Conversely, when the relationship between a concept and an attribute was inconsistent with participants’ implicit attitudes, it was deemed incongruent task. Participants tend to experience cognitive conflict when making this type of judgment, and so their reaction time would likely be longer. In both cases, the speed of reaction was taken to be a measure of the associative strength between the concept and the attribute (Williams and Themanson, 2011).

The advantages of the IAT are as follows: First, it minimizes the issue of social expectations that often affects self-report research (Fazio et al., 1995; Wittenbrink et al., 1997). Second, the IAT can facilitate the analysis of implicit associations by effectively excluding explicit familiarity with the concepts (Dasgupta et al., 2003).

### Method

#### Participants

Among 84 college students (mean age = 18.61 ± 0.98), all right-handed and not red-green colorblind, none took part in a similar psychological experiment before. To enhance the response rate, pre-paid envelopes were provided along with a small gift. 40 Taekwondo athletes (29 of whom were male) and 44 other college students (37 males) took part in the study. There are 16 national second-grade athletes and 24 national first-grade athletes. Their professional sports training age ranges from seven to ten years. Moreover, they have good physical fitness, strong professional skills, and rich competition experience.

#### Materials and Procedure

The materials consisted of two dimensions: a concept dimension (including picture material) and an attribute dimension (including text material). These concept materials consisted of 20 photos of Taekwondo athletes wearing red uniforms and blue uniforms. At first, ten athletes were selected to take photos in red uniforms. After that, the RGB criterion of red (255, 0, 0) was applied to the photos. Next, through Photoshop software, the RGB criterion of blue (0, 0, 255) with the same brightness and saturation was used to fill the red uniform area in the photos. The HSL (hue, saturation, and lightness) criteria are as follows: Red (0, 240, and 120) and blue (160, 240, and 120). Finally, the photos were carefully revised; the image format is JPG file with a size of 129 kb. The purpose is to prevent athletes from random movement and changing expression. Besides, the attribute material was made up of the ten words used in Experiment 1, and all words were repeated twice.

The experiment used special software E-prime 2.0 with a 12.1-inch screen Lenovo Think pad X200 for the stimulus presentation and data logging. A fixation cross appeared for 500 ms in the center of the computer screen, and the image stimulus-rendering time was 3,000 ms. Within the 3,000 ms, participants who did not make a response went directly to the next trial. A 500-ms blank screen was presented between each trial. The test was divided into seven blocks, and it consisted of 110 trials. Each participant completed the test on their own according to the instructions, and all were tested separately. Blocks 1, 2, 3, 5, and 6 served as practice rounds, and Block 4 and Block 7 were the test rounds; Block 4 featured the congruent tasks. When the picture stimulus appeared as an athlete in a red uniform and the word appeared as an aggressive-related word, the participants would press the “F” key to react; when the picture stimulus appeared as an athlete in a blue uniform and the word appeared as an agreeable-related word, the participants would press the “J” key to react. In the congruent tasks, participants were asked to classify an athlete in red uniform and an aggressive-related word as well as an athlete in blue uniform and an agreeable-related word. In Block 7 (i.e., an incongruent task), on the contrary, participants were asked to classify an athlete in red uniform and agreeable-related words as well as an athlete in blue uniform and aggressive-related words. After the experiment, a color blindness test was performed. The subjects were asked whether they could guess the purpose of the experiment, while all the subjects said no. Finally, in order to control a sequence effect, Blocks 2, 3, and 4 were, respectively, switched with Blocks 5, 6, and 7 for half of the participants.

### Results and Discussion

Experiment 2’s data were analyzed according to standard procedures (Greenwald et al., 1998). We recorded reaction times below 300–300 ms and those above 3,000–3,000 ms, and we also discarded incorrect responses. Following the preliminary processing of the data, there were 84 participants in the experiment and 79 valid data sets were obtained.

The experiment had a Groups (college students vs. Taekwondo athletes) × Combination (compatible vs. incompatible) ANOVA to explore whether college students and Taekwondo athletes would make different associations between Color and Valence. The results showed a significant main effect of Groups but not Combination, *F*(1,77) = 11.62, *p* = 0.007 (*p* < 0.01), η^2^ = 0.06, which indicated that athletes were found to be faster in categorizing compatible task than college students. In addition, a significant interaction effect between Groups and Combination was significant, *F*(1,77) = 4.03, *p* = 0.041 (*p* < 0.05), η^2^ = 0.02, which indicated that athletes were faster in categorizing congruent task (*M* = 578.43 ms, SD = 128.03) than incongruent task (*M* = 601.24 ms, SD = 162.19), *t*(36) = −3.302, *p* = 0.001 (*p* < 0.01), and college students were faster in categorizing congruent task (*M* = 593.27 ms, SD = 142.75) than incongruent task (*M* = 612.15 ms, SD = 149.74), *t*(41) = 2.211, *p* = 0.027 (*p* < 0.05). The results showed that the athletes responded faster to compatible task compared with the college students, while the response of incompatible task was not different between the athletes and the college students.

The results not only confirmed the previous hypothesis but also revealed that athletes responded faster on congruent tasks than college students. The reason why athletes reacted faster was as follows: in a competitive context, athletes wearing red uniforms were regarded as more aggressive, which suggested that the Taekwondo athletes had a stronger awareness of the association of red–aggressiveness. Overall, in the IAT, both the Taekwondo athletes and the college students responded significantly slower during the incongruent tasks compared to the congruent tasks.

## Discussion

Results from the above two experiments supported the hypothesis that there is an implicit link between red (or blue) and aggressiveness (or agreeableness), which is in line with the findings of previous studies using relatively straightforward methodologies (e.g., Krenn, 2014, 2015). Implicit measures were applied in this study to explore the advantage of red. This methodological improvement made the red–aggressiveness association clearer. Another important contribution of the present study is that photos of athletes are used in the IAT experiment, which is closer to a real competitive context.

Experiment 1 used a modified Stroop task to present textual stimuli and found an implicit association between red and aggressiveness. This is in keeping with the views of Briki and Hue (2016), who found a strong association between red and dominance. There may be the following explanations. Firstly, it could be related to biological factors. In the animal world, red is often related to danger. Likewise, human beings possess a biologically engrained predisposition to associate red with aggressiveness. Secondly, although having a positive meaning for Chinese people in general, red is usually used to signify negative connotations in an educational context. For example, teachers use red pen to mark incorrect answer. Since the participants are all college students, red may arouse their negative association with aggressiveness. In addition, participants have no idea about the purpose of the experiment; thus, they showed little awareness of these red effects. This suggests the red–aggressiveness association appears to take place outside of participants’ conscious awareness (Elliot and Maier, 2007). Little and Hill (2007) suggested that the dominance of red can be influenced by hue information in the stimuli. Therefore, the present study left the question of which type of red is most strongly connected to aggressiveness. To find the exact connection between red and aggressiveness, it would be helpful to conduct various experiments in the future. For instance, hue is held constant while lightness and chroma are systematically varied in different ways. In line with previous studies (Pravossoudovitch et al., 2014; Mentzel et al., 2017), no gender difference was found in this study. From an evolutionary point of view, humans are born to relate red with the danger. The implicit cognition of the red–aggressiveness association was strong for both male and female. Therefore, it can be inferred that aggressiveness is a characteristic not only of male but also of female, and there is no significant difference to some extent. Future research can continue to explore whether there is gender difference in implicit cognition between male and female.

Meanwhile, similar to the results from the United States, Germany, and Turkey’s participants (Garth and Collado, 1921; Gesche, 1927; Choungourian, 1968), the present study also confirms that participants are faster in response to the blue–agreeableness association, which suggests that blue is associated with the notion of positive and agreeable in Chinese culture. More recently, Mentzel et al. (2017) also found an implicit link between red and dominance through a Stroop task, but they failed to find an implicit connection between blue and rest. A reason for this difference may be that the concepts of agreeableness and aggressiveness used in the present study have opposite meanings, while the contrasting meanings between dominance and rest are not obvious in Mentzel’s study. What is more, Curby (2016) found that in men’s freestyle wrestling, wearing a blue uniform was significantly associated with winning. This led the doubt whether there is blue uniform effect. Further studies are needed to verify it.

In Experiment 2, Taekwondo athletes felt a stronger implicit association between red and aggressiveness than college students. Taekwondo athletes often participated in sport competitions, whereas college students are seldom exposed to competitive sports. Thus, they may react to competitive context differently. Cooper (1969) showed that athletes have a higher achievement motivation as well as a higher self-confidence and aggressiveness than others. Kirkcaldy (1982) also found that elite athletes are more extroverted and aggressive. From this point of view, athletes may have a more aggressive and impulsive personality than college students. Sometimes, red is commonly linked to love and romance in the affiliation context. Future research may explore red’s meaning among athletes in other contexts.

Moreover, Dreiskaemper et al. (2013) found that athletes in red jersey would have significantly higher heart rates and significantly higher pre-contest values on the strength test, but it did not influence the results. This suggested that the influence of red on psychological functioning is as pervasive as it is subtle and provocative. Continuous researches are needed to clarify the impact of red on athletes and opponents. There is no difference between athletes and college students in the incongruent task. This indicates that little close association exists between agreeableness-related words and athletes wearing red. This provided indirect support for the red–aggressiveness association. Participants were more likely to think that athletes wearing red uniforms were more aggressive. As mentioned above, social cultural elements might affect red and its associations. The present study confirmed the association between red and aggressiveness among Chinese people, which is in accord with the research carried out in the West.

The study also offers a wide range of implications for daily life. For example, red, be it associated with dangers and mistakes, could activate an avoidance motivation. It has been shown to make people more vigilant and risk-averse; hence, red is not the main color used in hospital wards. What then is the most attractive color for advertisement? There are two cases. If the advertisements aim at warning, red is preferable. On the contrary, if concerning environmental protection, blue is a better choice. Furthermore, associated with peace and tranquility, blue is likely to activate an approach motivation and thus is usually used to encourage people to create new things.

The meaning of color is used not only in daily life but also in the competitive context. If there is an association between red and aggressiveness, the influence of the association on the referee’s penalty would be automatic and unconscious. Since the referee’s penalty will be affected by the red uniform, it will have an adverse effect on the fairness of the competition. When the referees’ self-control strength was low, the association of red–aggressiveness would have more influence on the referee’s penalty. Conversely, when referees’ self-control strength was high, the influence of the red–aggressiveness association on the referee’s penalty would be weakened. Bertrams et al. (2015) found that red had a negative effect on performance of participants with ego depletion, but hardly or not at all among control groups. This reminds us that referees should take some measures (e.g., glucose intake, enough rest, and taking exercise) to enhance self-control in order to prevent themselves from a red–aggressiveness association. In doing so, it may contribute to achieving fairness in competition and equal opportunities for each athlete to win, irrespective of uniform color.

In conclusion, although this study examined the association between red and aggressiveness, the effect size of the study is comparatively weak. Future researches are needed to explore the association between red and aggressiveness. In Experiment 2, the athletes were not in the same level; this may also have an impact on the results. Additionally, Krenn (2014) found that red uniform may affect the referee’s penalty. However, only college students and athletes are selected as participants in the present study. Future study may consider recruiting referees as participants. In terms of experimental material color, although blue and red were selected, which are commonly used as contrast colors in competitive sports, a more professional spectrophotometer could be considered to adjust brightness and chroma in future study. At present, the mechanism of the red–aggressiveness association is not clear. Therefore, future research should explore the brain mechanism of the association between red and aggressiveness.

## Conclusion

This study showed an implicit association between red and aggressiveness, as well as an association between blue and agreeableness, yielding an evidence for a link between colors and meanings. What is more, athletes were found to have shorter response times than college students regarding the association of red–aggressiveness.

## Data Availability Statement

The raw data supporting the conclusions of this article will be made available by the authors, without undue reservation.

## Ethics Statement

The studies involving human participants were reviewed and approved by WSU Medical Ethics Committee. The patients/participants provided their written informed consent to participate in this study. Written informed consent was obtained from the individual(s) for the publication of any potentially identifiable images or data included in this article.

## Author Contributions

XH was responsible for the design of the study and the supervision, planning, and feedback on the written article. LG was responsible for data collection and analysis and for writing the first draft of the article. YZ assisted in the experiment. All authors have approved the manuscript and given consent for its submission and subsequent publication.

## Conflict of Interest

The authors declare that the research was conducted in the absence of any commercial or financial relationships that could be construed as a potential conflict of interest.
